# Multivariate Analysis of the Influence of Microfiltration and Pasteurisation on the Quality of Beer during Its Shelf Life

**DOI:** 10.3390/foods13010122

**Published:** 2023-12-29

**Authors:** Ana Carolina de Lima, Luciana R. Brandao, Bruno G. Botelho, Carlos A. Rosa, Laura Aceña, Montserrat Mestres, Ricard Boqué

**Affiliations:** 1 Department of Analytical Chemistry and Organic Chemistry, Universitat Rovira i Virgili, Campus Sescelades, 43007 Tarragona, Catalonia, Spain; a_carolinalima@hotmail.com (A.C.d.L.); laura.acena@urv.cat (L.A.); montserrat.mestres@urv.cat (M.M.); 2 Laboratório da Cerveja Ltda, Nova Lima 34007-756, Minas Gerais, Brazil; biolubrandao@yahoo.com.br; 3 Department of Chemistry, Universidade Federal de Minas Gerais, Campus Pampulha, Belo Horizonte 31270-901, Minas Gerais, Brazil; botelhobrunog@gmail.com; 4 Department of Microbiology, Universidade Federal de Minas Gerais, Campus Pampulha, Belo Horizonte 31270-901, Minas Gerais, Brazil; carlrosa@icb.ufmg.br

**Keywords:** microfiltration, pasteurisation, forced ageing, microbiological stability, colloidal stability, flavour stability

## Abstract

Gas chromatography–mass spectrometry (GC–MS), physicochemical and microbiological analyses, sensory descriptive evaluation, and multivariate analyses were applied to evaluate the efficiencies of microfiltration and pasteurization processes during the shelf life of beer. Samples of microfiltered and pasteurised beer were divided into fresh and aged groups. A forced ageing process, which consisted of storing fresh samples at 55° C for 6 days in an incubator and then keeping them under ambient conditions prior to analysis, was applied. Physicochemical analysis showed that both microfiltered and pasteurised samples had a slight variation in apparent extract, pH, and bitterness. The samples that underwent heat treatment had lower colour values compared with those that were microfiltered. Chromatographic peak areas of vicinal diketones increased in both fresh and aged samples. The results of the microbiological analysis revealed spoilage lactic acid bacteria (*Lactobacillus*) and yeasts (*Saccharomyces* and *non-Saccharomyces)* in fresh microfiltered samples. In the GC–MS analysis, furfural, considered by many authors as a heat indicator, was detected only in samples that underwent forced ageing and not in samples that were subjected to thermal pasteurisation. Finally, sensory analysis found differences in the organoleptic properties of fresh microfiltered samples compared with the rest of the samples.

## 1. Introduction

Beer is an alcoholic beverage obtained from the fermentation of sugars (mainly maltose) extracted from barley malt during mashing. The quality and shelf life of beer is mainly determined by its appearance, aroma, flavour and texture, and are conditioned by its microbiological, colloidal and flavour stability [1,2]. To achieve and maintain beer stability, it is necessary to eliminate any form of biological contamination in the brewing process [3]. The main cause of beer spoilage in a brewery is organisms in the air, soil, water, raw materials, malt, pitching yeast, brewery equipment and machinery. According to Spedding [4], the spoilage caused by microorganisms includes turbidity, haze formation, ‘rope’ formation, over-attenuation, gushing, souring of wort and beer and production of different off-flavours.

Due to globalisation and the increasing exportation of beer, shelf-life problems have become an important issue for breweries. Despite this, some beer parameters, such as alcohol content, low pH, low nutrients and reduced oxygen content, make it an inherently stable product. To achieve microbial stability and guarantee product quality during its shelf life, breweries must adopt measures such as microfiltration and/or pasteurisation [5,6]. In some craft breweries, beer is nowadays microfiltered to achieve microbiological and colloidal stability. On the other hand, the brewing industry applies thermal treatments such as pasteurisation at the end of the process to guarantee microbiological stability and extend the shelf life of the product. 

The purpose of filtering beers is to clarify the product by removing yeast cells, some colloidal particles and hop residues without causing any significant change in the quality of the final product. On the contrary, the pasteurisation of beer aims to reduce the microbial and enzymatic activity found in the final product, considerably extending its shelf life, although at the expense of changes in the sensory profile.

Microfiltration is a membrane filtration process that consists of flowing a particle-rich liquid through a polymeric membrane designed to have pore diameters of 0.2–1.3 µm [7,8]. The membranes can retain yeast cells and beer spoilage bacteria that are 5–10 µm and 0.45 µm in size, respectively, hence achieving microbiological stability [7]. Moreover, colloidal stability can be achieved by removing other large particles such as polyphenols and protein flakes, which appear as a “chill haze” at low temperatures in the clarified beer. According to dos Santos et al. [8], microfiltration is used for the filtration and pasteurisation of beers, properly retaining yeast cells, spoilage bacteria, polyphenols and protein flakes, hence avoiding significant changes in the sensory characteristics. It has been shown that microfiltration can provide flavour stability and at least six months of shelf life, avoiding the costs associated with conventional pasteurisation [8].

Pasteurisation is a thermal process that aims to inactivate yeast cells and spoilage microorganisms. Pasteurisation allows the beer to remain stable for a longer period, thus increasing its shelf life. The intensity of this thermal process is measured in pasteurisation units (PUs), where 1 PU is defined as 1 min treatment at 60 °C [9]. Bottled/canned beers are generally pasteurised in pasteurisation tunnels, which consist of progressively hotter zones, holding zones and progressively cooler zones to ensure microbiological control of the product and maintain most of its nutritional value and sensory properties [8,10]. According to the literature, the application of a minimum of 15 PUs is sufficient to achieve practical sterility against brewer’s yeast, *Pediococcus* sp., *Lactobacillus* sp., and wild yeast [6,9]. 

In general, both microfiltration and pasteurisation have advantages and disadvantages. The advantages of microfiltration include eliminating the use of filter aids, reducing beer losses, high solid handling capacity, and replacing thermal pasteurisation for better product quality and cost savings [8,11]. However, some authors argue that, on a large scale, this process still presents considerable technical and economic barriers, including severe membrane fouling, quality variation across different beer brands in the same membrane system and inconsistent quality of products filtered through a system of membranes [7,8,11]. However, breweries are using microfiltration more and more, with quite satisfactory results. Regarding pasteurisation, despite favouring microbiological stability and extending beer shelf life, this process presents some disadvantages. According to Stuart and Priest [12], pasteurisation accelerates colloidal haze formation and breaks the stability between high-molecular-weight proteins and polyphenols. Additionally, pasteurisation is one of the main causes of changes in the sensory properties that affect the qualitative characteristics of beer [13].

Since the thermal process can have a negative impact on beer quality, some studies have reported the effect of this process on different characteristics associated with beer quality. Cao et al. [1] analysed the impact of different pasteurisation units (2 PUs, 8 PUs and 14 PUs) on beer ageing and flavour stability during a 6-month storage period at room temperature. The key factors examined were colour, the thiobarbituric acid (TBA) index, 1,1-diphenyl-2-picrylhydrazyl (DPPH) radical scavenging activity, bitterness, total polyphenol composition, the concentration of beer volatile compounds and 5-hydroxymethyl furfural (5-HMF). The authors concluded that statistically speaking, samples with 14 PUs exhibited the greatest flavour change and the highest degree of damage. 

As the beer market is in steady expansion and consumers are more concerned about product quality, the brewing industry needs to offer a product that can maintain stability and the best organoleptic characteristics during its shelf life. In this study, the influence of microfiltration and pasteurisation on the quality and stability of beer during its shelf life was compared in fresh and forced-aged beers. This research aimed to evaluate the efficiency of both processes during beer shelf life, correlating the main parameters that influence the sensorial quality and stability of the product, such as microbiological, colloidal and flavour stability, using multivariate analysis. 

## 2. Materials and Methods

### 2.1. Beer Samples

In total, 136 samples of a commercial Lager beer from the same batch, with an alcohol content of 4.8% *v*/*v* and packaged in glass bottles, were delivered directly from a local brewery of Belo Horizonte (Brazil), in the freshest possible condition. Of these, 68 were microfiltered and 68 were pasteurised. The microfiltered samples were taken off the bottling line after the capping phase and the pasteurised samples were kept on the line to pass through the pasteurisation tunnel. For microfiltration, 0.22/0.45/1.0 µm membranes were used to ensure microbiological, colloidal and taste stability. The pasteurisation process was carried out in a pasteurisation tunnel for a total of 40 min, where samples were heated to 62 °C and then cooled to the refrigeration temperature (4 °C). The 136 samples were divided into 68 fresh and 68 aged samples, and, in both cases, there were 34 microfiltered beers and 34 pasteurised beers. This information is summarized in Table 1. The forced ageing process was performed by storing fresh samples at 55 °C for 6 days in an incubator (Incubator B.O.D NL-161.01 New Lab—Piracicaba, São Paulo, Brazil) and then keeping them under ambient conditions prior to analysis.

### 2.2. Physicochemical Analysis

To better understand the influence of the microfiltration and pasteurisation processes on the quality of the product during its shelf life, physicochemical analyses were carried out using the official methodology indicated in Analytica Brewery Convention [14]. The parameters analysed were apparent extract (9.4), pH (9.35), alcohol (9.2.1), colour (9.6), IBU–International Bitterness Unit (9.8) and vicinal diketones (9.24.1) [14]. Before the analyses, all samples were degassed for 15 min via ultrasonication.

### 2.3. Microbiological Analysis

Microbiological analysis was conducted according to the procedures described in Section 4.0 of The European Brewery Convention–Analytica Microbiologica for detecting contaminants in beer [15]. All samples were prepared and analysed in duplicate.

#### 2.3.1. Culture Media

For the detection of non-*Saccharomyces* yeast, Lysine Agar (4.2.6) was used because the presence of lysine favours the development of wild yeast that does not belong to the *Saccharomyces* genus. Yeast Medium (4.2.5.1), which contains malt extract, glucose and peptone with CuSO_4_, was also used for the detection of *Saccharomyces* wild yeast because the presence of Cu inhibits the growth of culture yeasts and thus allows the growth of wild yeasts, including *Saccharomyces* [16]. For the isolation of aerobic and anaerobic bacteria, WLD (Wallerstein Laboratory Differential Medium) with cycloheximide was used to inhibit yeast growth. The medium was prepared according to the manufacturer’s instruction (Difco^TM^, Le Pont de Claix, France) by suspending 80 g of the powder in 1 L of purified water, mixing thoroughly, heating with frequent stirring, boiling for 1 min to completely dissolve the powder and autoclaving at 121 °C for 15 min. Cycloheximide was used to inhibit yeast growth. The colour of the solution was blue to greenish blue. 

All culture media were prepared in the laboratory, sterilized in an autoclave and distributed on a plate under sterile conditions in a laminar flow hood.

#### 2.3.2. Sample Preparation, Inoculation and Plate Reading

After going through the forced ageing process, the beer was kept in the dark at a temperature of 20 ± 2 °C. Prior to analysis, beer containers were sprayed with alcohol before opening. All analyses were performed under sterile conditions in a vertical laminar flow hood. Direct sowing of beer was used on the culture medium, according to the procedures described in the European Brewery Convention–Analytica Microbiologica, Section 2.0 [15]. Seeding was performed using 0.1 mL of sample per plate, all in duplicate. All the inoculated culture media were incubated at a controlled temperature and atmosphere. Incubation is a necessary step between the inoculation of the culture medium and the examination of the cells grown on the plates. Aerobic incubation, in atmospheric air, was used for the detection of non-*Saccharomyces* yeast, (Lysine Agar), *Saccharomyces* wild yeast (YM) and the aerobic count of bacteria (WL). The aerobic incubation condition was 5 days for all samples, and the temperature used for the detection of non-*Saccharomyces* and *Saccharomyces* wild yeast was 27 ± 1 °C, while that for the aerobic bacteria was 37 ± 1 °C. For anaerobic bacterial count (WL), an air-tight jar with an anaerobic generator was used and the incubation process took 5 days at a temperature of 37 ± 1 °C.

At the end of the incubation period, the plates were inspected by direct visual examination. The colonies of yeast cells were identified and counted. The Gram staining (2.3.6.1) method described in The European Brewery Convention–Analytica Microbiologica [15] was used for the differentiation of Gram-positive and Gram-negative bacteria. Gram staining depends on differences in the cell wall structure. The results may show a permanent blue or violet colour, indicating Gram-positive bacteria, or a red or pink colour, indicating a Gram-negative bacteria. Once the Gram staining test had been performed, the catalase test (2.3.7) for differentiating catalase-positive from catalase-negative bacteria was conducted using a 3% *v*/*v* hydrogen peroxide solution [15].

### 2.4. Gas Chromatography Analysis

#### 2.4.1. Sample Preparation

The optimal conditions that allowed the extraction of the largest number and intensity of odorants were achieved by placing 50 mL of sample into a 250 mL round bottom flask, with 50 µL of a solution of 400 mg L^−1^ of 5-nonanol in distilled water as an internal standard (final concentration of 0.4 mg L^−1^). Samples were distilled until 10 mL of distilled solution was obtained and then injected into the GC–MS. All samples were degassed by ultrasonication prior to the analysis and were analysed in quintuplicate.

#### 2.4.2. Gas Chromatography Analysis

Samples were analysed with GC–MS equipment, model Shimadzu^®^ GCMS-QP2010 (Duisburg, Germany) with an automatic injector. It was composed of an HP 7690 gas chromatograph (Waldbronn, Germany) and a 5977B HES mass spectrometric detector (Santa Clara, CA, USA) equipped with a turbo pump and a high-efficiency ion source. To carry out the chromatographic separations, an Elite-WAX (Shelton, CT, USA) (30 m × 0.32 mm i.d., 0.25 µm film thickness) fused silica capillary column was employed. A 1-µL volume of the distilled solution was injected in splitless mode at a temperature of 150 °C. The carrier gas used was Helium. The oven temperature was programmed as follows: the initial temperature was 30 °C; after 10 min it was raised from 15 °C min^−1^ to 150 °C and held for 2 min; finally, the temperature was raised at a rate of 15 °C min^−1^ to 220 °C and held for 5 min. The mass spectra were recorded by electronic impact (EI) ionization at 70 eV with a temperature of 230 °C in the ion source and 150 °C in the mass quadrupole. The mass range analysed in the scan mode was from 25 m/z to 200 m/z.

#### 2.4.3. Compound Identification

The odorants detected were identified using the Automatic Mass Spectral Deconvolution and Identification System (AMDIS) using a library of mass spectral database (NIST MS Search Library version 2.3) by comparing with reference substances based on the retention index (RI) in the Flavornet website [17].

### 2.5. Sensory Descriptive Analysis 

Beer samples were sensory-evaluated by a panel of 11 trained assessors aged between 30 and 50 years old, following the methodology and statistical analysis described in Sensory Analysis–Description Analysis (13.10) of the European Brewery Convention [14]. Four numbered samples: 01 Fresh Microfiltered, 01 Fresh Pasteurised, 01 Aged Microfiltered and 01 Aged Pasteurised were presented to the panellists. Each assessment rated the intensity of descriptors on a 10-point scale (a score of 1 meant that the aspect analysed was slight, whereas a score of 10 meant that the aspect analysed was very intense). All samples were kept at 10 °C for 48 h prior to the analysis. The attributes evaluated are described in Table 2.

### 2.6. Chemometric Analysis

The chromatographic, physicochemical and sensory information collected on the samples was used to discriminate beer samples using chemometric classification and prediction methods. The data obtained were organized in a matrix X (20 × 70), where samples were placed in the rows and the columns represented the results of the integrated peak area of the 12 identified compounds, the physicochemical results of the six analyses and the results of the 52 parameters evaluated in the sensory descriptive test. Since the differences in magnitude between the data were significant and could affect the analysis, the values of the data matrix were pre-processed by dividing each value in the matrix by the total sum of the values contained in the array before the chemometric analysis. Then, the data were autoscaled (i.e., mean centred and standardized to unit variance).

For a preliminary visualization of the data, Principal Component Analysis (PCA) was used, as this exploratory method may help to explain the correlation between variables, reveal the main differences between microfiltered and pasteurised samples and point out outlier samples. 

Partial Least Squares (PLS2) regression was used to correlate data derived from instruments (GC–MS and physicochemical results) to the sensory descriptors. The data obtained from these analyses were organized in a matrix X (20 × 22), where samples were placed in the rows and variables in the columns. Variables represent the integrated peak areas of the identified compounds with the exception of ethanol, the physicochemical results (pH, VDK, colour, IBU and apparent extract), and the results of the sensory descriptive test (foam colour, turbidity, foam size, foam quality, lactic acid, biscuit, bread and acidity). Finally, Partial Least Squares Discriminant Analysis (PLS-DA) was applied to classify the samples according to the conservation process applied, helping to understand the main parameters/compounds that make this differentiation possible. All the analyses were performed using the MATLAB R2020a software (MathWorks, Natick, MA, USA) supported by the PLS Toolbox 5.2.2 (Eigenvectors Research Inc., Manson, WA, USA).

## 3. Results

### 3.1. Physicochemical Analysis

**Figure 1:**
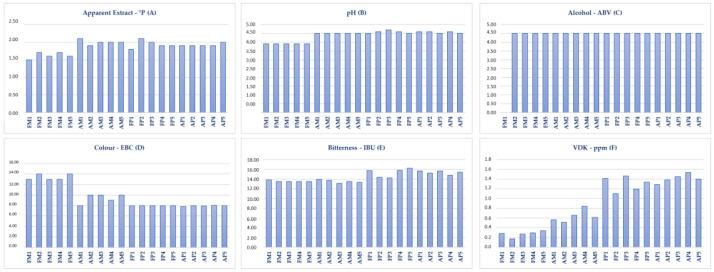
FM: Fresh Microfiltered; AM: Aged Microfiltered; FP: Fresh Pasteurised; AP: Aged Pasteurised.

Physicochemical analysis was performed to better understand the influence of the microfiltration and pasteurisation processes on the quality of the final product during its shelf life. Figure 1 shows the physicochemical parameters studied.

From the analysis of the physicochemical results, we observed that the values of apparent extract (A) and pH (B) of fresh microfiltered samples were slightly lower than those of the other samples. The alcohol (C) remained constant in all samples. Beer colour (D) decreased after heat treatment and this decrease was less affected by the forced-aged process than by the pasteurisation process. IBU (E) showed a slight variation between microfiltered and pasteurised samples. Finally, VDK (F) showed a significant increase after heat treatment, especially between the fresh microfiltered and the fresh pasteurised samples. Table S1 in the Supplementary Materials shows the ANOVA (Analysis of Variance) results for the physicochemical analysis of the different samples. The table shows that the samples of pasteurised beer, both fresh and aged, did not present significant differences between them for any parameter analysed, with the exception of pH where the AP samples showed similarities with the FP and AM samples. The microfiltered samples showed significant differences in apparent extract, colour, pH and VDK. Despite the slight variation in the apparent extract of AM samples shown in Figure 1, the ANOVA results indicate that AM samples and both pasteurised samples (FP and AP) are similar in terms of apparent extract.

### 3.2. Microbiological Analysis

Microbiological analyses were performed to assess the efficiency of both microfiltration and pasteurisation processes to determine the microbiological stability of the product. For this, the detection of *Saccharomyces* and non-*Saccharomyces* yeasts, isolation of aerobic and anaerobic bacteria, Gram staining and catalase tests were performed. The results of the microbiological analyses are shown in Table 3.

Based on the results of the microbiological analysis (Table 3), beer spoilage bacteria and yeasts were detected only in the microfiltered fresh beer samples. The plates of YM and Lysine were analysed visually to count the colonies and then evaluated microscopically. Non-*Saccharomyces* colonies were detected on some YM plates. All fresh microfiltered samples showed colony-forming units greater than 300 colonies/0.1 mL for both aerobic and anaerobic bacteria. All the bacteria detected were Gram-positive and catalase-negative, with the most predominant being the lactic acid bacteria, *Lactobacillus* and *Pediococcus* [18]. Microscopic analysis of the samples suggests that the bacteria detected could be from the genera *Lactobacillus*. The other samples that went through thermal treatment, both aged and pasteurised, did not show any bacterial or yeast spoilage.

### 3.3. GC–MS Analysis

The results of the GC–MS analysis showed the evolution of the volatile compounds present in both fresh and aged samples of microfiltered and pasteurised beers. Table 4 shows the 12 volatile compounds identified in the microfiltered and pasteurised beers.

The results of the GC–MS analysis showed the presence of furfural and 2,3-butanediol in the AM samples only. Methyl benzoate and benzoic acid were absent in the same samples.

### 3.4. Sensory Descriptive Analysis 

**Figure 2:**
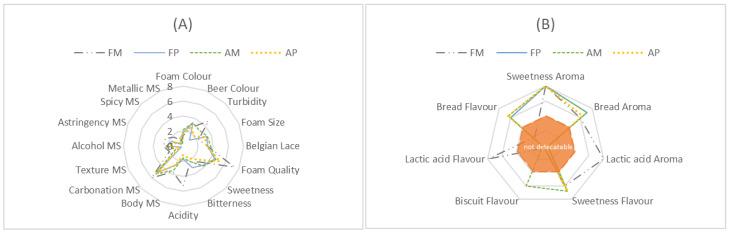
Overall impression of sensory analysis. Visual description, mouth sensation and taste description (**A**); aroma and flavour description (**B**). FM, fresh microfiltered; FP, fresh pasteurised; AM, aged microfiltered; AP, aged pasteurised; MS, mouthfeel sensations.

Data from the descriptive analysis were evaluated based on the conservation process imposed, microfiltration or pasteurisation, and its efficiency regarding organoleptic quality and stability of the product during its shelf life (fresh and aged). Figure 2 shows the overall impression of the sensory analysis.

As can be seen in Figure 2, fresh microfiltered (FM) samples showed some differences in all descriptive analyses conducted by the panellists. For visual description (Figure 2A), FM was qualified as the sample with the best foam quality and more turbid. For aroma description (Figure 2B), FM showed three compounds, including lactic acid, sulphurous and phenolic, that were not perceived with the same intensity in the other samples. For flavour description (Figure 2B), lactic acid and phenolic flavours were perceived more strongly in FM samples. Finally, for taste analysis and mouthfeel sensation (Figure 2A), panellists described the FM samples as more acidic, spicier and more astringent than the others. 

### 3.5. Chemometric Analysis 

**Figure 3:**
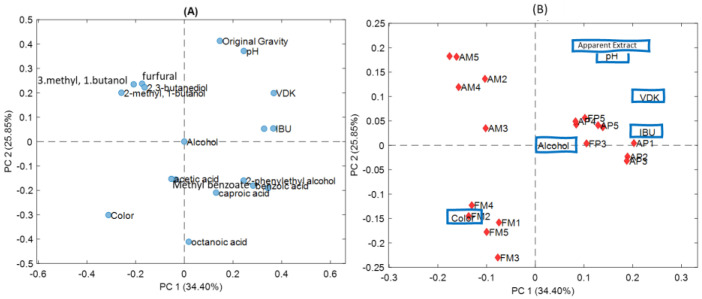
(**A**) Loading PCA plot of the chromatographic data and physicochemical results for both microfiltered and pasteurised samples; (**B**) PCA biplot of the chromatographic data and physicochemical results of samples and physicochemical results: Apparent extract, pH, Alcohol, Colour, IBU, VDK. FM: Fresh Microfiltered; AM: Aged Microfiltered; FP: Fresh Pasteurised; AP: Aged Pasteurised.

Principal Component Analysis (PCA) was applied to the chromatographic data matrix and the physicochemical results. Figure 3 shows the loading plot (A) and the biplot (B) of the chromatographic data and physicochemical results (scores and loadings) for the first two principal components of the PCA, which explains around 60% of the total variance in the data. In the plot, the correlation between the physicochemical results and the fresh and aged samples of both microfiltered and pasteurised conservation processes can be observed. 

Although the sample size (20 beers) was certainly not large, at least some trends were detected at an exploratory level. Figure 3A shows the loading PCA plot. It can be observed that furfural (4) and 2,3-butanediol (6) are positively correlated with the AM samples. Methyl benzoate (7) and benzoic acid (12) had a positive loading on PC1, suggesting that it was positively correlated with the pasteurised samples and negatively correlated with the microfiltered samples. Octanoic acid (11), which showed a decrease in the peak area in pasteurised and forced-aged samples, had a negative loading in PC2, suggesting that it was positively correlated with fresh microfiltered samples. Furfural (4), which is considered by some authors, such as Aron and Shellman [19] and Saison et al. [20], as an indicator of thermal treatment, had a positive loading on PC2, suggesting that it was positively correlated with the samples that went through heat treatment. 

The biplot in Figure 3B shows that the group of microfiltered and pasteurised samples, both fresh and forced-aged, have opposite correlations in PC1. In the same figure, we observe that the pasteurised samples (FP and AP) are more grouped than the microfiltered ones (FM and AM), which show more dispersion. 

Table S2 in the Supplementary Materials shows the ANOVA results for the GC–MS analysis of the different samples. It can be seen that, with the exception of the AM samples where these compounds could not be identified, all the samples showed some similarities based on 2-methyl 1-butanol (2), 3-methyl, 1-butanol (3) methyl benzoate (7) and 2-phenylethyl alcohol (10). For some compounds such as acetic acid (5) and benzoic acid (12), samples did not show any significant differences, regardless of whether they were microfiltered or pasteurised. On the other hand, octanoic acid (11) showed differences in the microfiltered and pasteurised samples.

By analysing the correlation between physicochemical results and samples, we observed that colour had a negative loading in PC1, suggesting that it was positively correlated with FM samples and negatively correlated with AM samples. IBU, VDK, Original gravity and pH had a positive loading in PC1, indicating that they were positively correlated with both fresh and aged pasteurised samples. Alcohol appeared in the centre of the plot, indicating that it had a low influence on the PCA model. We also observed, in the same figure, that PC1 explained the difference between microfiltered and pasteurised samples while PC2 seemed to explain the difference between the samples that underwent heat treatment AM (forced ageing with temperature), FP (pasteurisation process) and AP (pasteurisation and forced ageing processes) and those that did not, such as FM (microfiltration process). 

After the preliminary PCA, we applied a regression model to observe the correlation between instrumental data and sensory descriptors. For this, PLS2 was applied by regressing the instrumental **X** data matrix (GC–MS and physicochemical results) to the **Y**-matrix of sensory descriptors. The model was leave-one-out cross-validated. Figure S2 in the Supplementary Materials shows the correlation loadings of the PLS2 model with all X and Y variables (GC–MS, physicochemical results and sensory analysis). The aim was to reduce the number of variables in the data matrix, keeping only those that have more weight when analysing the efficiency of the microfiltration and pasteurisation processes in relation to the sensory quality of the final product during its shelf life. Table 5 shows the variables with more influence on the efficiency of the microfiltration and pasteurisation processes in relation to the sensory quality.

**Figure 4:**
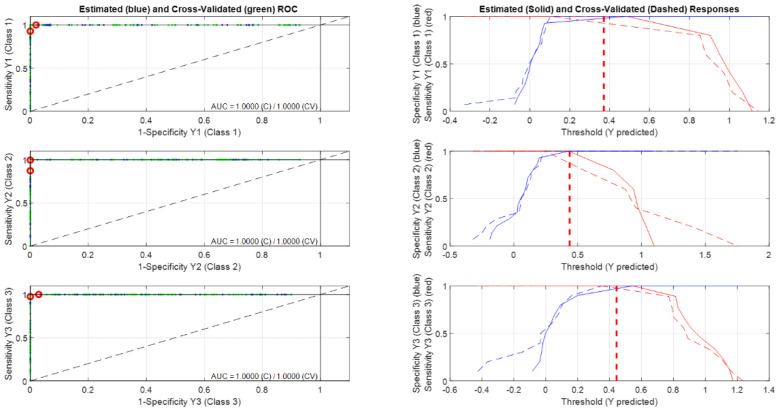
Threshold /ROC plots for the classification model with three classes: Class 1: FM; Class 2: (AM); and Class 3 (FP + AP).

Finally, to better understand the effect of the studied parameters on beer quality during ageing based on the conservation process (microfiltration and pasteurisation). After the selection of the most influential variables, Partial Least Squares Discriminant Analysis (PLS-DA) was applied to classify samples according to the conservation process applied and to ageing (fresh or aged). The aim was to understand how the samples are classified and what the main parameters/compounds that make this differentiation possible are. First, PLS-DA was applied to classify the four classes: Class 01 (FM samples), Class 02 (AM samples), Class 03 (FP samples) and Class 4 (AP samples). In this classification model, the pasteurised samples were not distinguished from each other (results not shown), hence we combined FP and AP samples and performed a classification model with three classes: Class 01 (FM samples), Class 02 (AM samples) and Class 03 (FP and AP samples). The model was leave-one-out cross-validated, and the optimal number of LVs was determined based on the percentage of correctly classified samples for the cross-validation set. Figure 4 shows the Threshold/ROC plots for the classification model with three classes. It can be observed that all samples in the three classes are correctly classified. 

**Figure 5:**
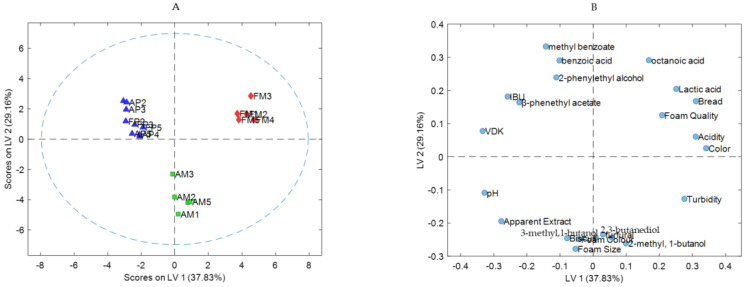
Score plot (**A**) and loading plot (**B**) of the PLS-DA classification model with three classes: Class 1: FM; Class 2: (AM); and Class 3 (FP + AP).

Finally, Figure 5 shows the score and loading plots of the PLS-DA classification model.

By observing the plots in Figure 5, it can be seen that 2,3-butanediol and furfural are positively associated with the AM samples. Additionally, the same loading plot shows that physicochemical parameters such as apparent extract and pH, and the sensory visual and aromatic descriptors like turbidity, foam size and colour, and biscuit, respectively, are associated with the forced-aged microfiltered samples.

## 4. Discussion

Although the pasteurisation process increases the colour of beer due to the Maillard reactions, in this study, the pasteurised samples, both fresh and aged, presented lower colour values than the microfiltered ones. This could be explained as a result of contamination in the microfiltered samples due to spoilage. Since the pasteurised samples went through thermal treatment just before the bottling process, the microorganisms that spoiled the beer were inactivated before they could produce any substantial changes in the physicochemical characteristics of the product. The AM samples also went through heat treatment to force ageing, but unlike the pasteurised ones, this process was applied a few days after bottling, which may have contributed to changes in some physicochemical parameters such as colour, IBU and VDK. On the contrary, the FM samples did not receive any heat treatment, which allowed the spoilage microorganisms present in those beers to act by altering the physicochemical and sensory parameters to a greater degree than the AM samples. Analysing the correlation loadings of the PLS2 model (Figure S1 in the Supplementary Materials) shows that colour is directly correlated with lactic acid (0.80), acidity (0.86) and turbidity (0.64) and inversely correlated with pH (−0.95), VDK (−0.86), IBU (−0.66) and apparent extract (−0.81). The physicochemical results show that the FM samples have lower values for VDK, IBU and apparent extract than the other samples. This is also true for the AM samples, but to a lesser degree. Additionally, FM samples are directly associated with the sensory attributes, lactic acid, acidity and turbidity. All these parameters are associated in some way with spoilage contamination in beer. For the physicochemical results of colour (Figure 1), although the FM samples had higher colour values than the other samples; however, in the descriptive sensory test, the panellists could not detect this difference visually and scored almost all the samples equally. 

Vicinal diketones (VDKs) are produced by yeasts during fermentation in the presence of oxygen and enzymatically consumed in the absence of oxygen. The most significant VDKs for the beer production process are diacetyl (2,3-butanedione) and 2,3-pentanedione, due to their low flavour thresholds of 0.1 and 0.9 ppm, respectively, giving beer butter/butterscotch and toffee-like flavours [21,22]. Diacetyl, which has a significant effect on flavour, aroma and drinkability, is re-assimilated and reduced via acetoin in 2,3-butanediol, which has a flavour threshold of around 4500 ppm [22,23,24,25]. Based on the GC–MS results (Table 4), 2,3-butanediol could be detected only in the aged microfiltered (AM) samples. Because of its higher flavour threshold, the cream/buttery odour of this compound could not be detected by the panellists during the descriptive analysis, as shown in Figure 2A. Despite the concentration of VDKs in the pasteurised samples being almost six times higher than in the microfiltered samples, neither diacetyl nor 2,3-pentanodione could be detected instrumentally or sensorially in any sample. The presence of VDKs could be explained as the result of yeast removal or inactivation before the maturation stage [26]. 

Despite being considered an unfavourable environment, a limited number of bacteria and yeasts are capable of growing and spoiling beer, especially if it is unpasteurised or microfiltered beer [27,28,29]. The microbiological analysis detected the presence of wild yeast (*Saccharomyces* and non-*Saccharomyces*) and Gram-positive bacteria (catalase-negative) spoilages in the fresh microfiltered samples. The ideal microbiological situation for a beer is to have only one species of organism present in the product, namely the culture yeast pitched into the wort. Although these types of yeasts are desirable in some types of beer such as lambic and gueuze beer, wild yeasts in lager beer are considered spoilage organisms and must be avoided. *Saccharomyces diastaticus* is considered the main *Saccharomyces* spoiler in beer [30]. Contamination of beer by wild yeasts is manifested by the fermentation of residual carbohydrates, including dextrins and starch, by haze formation, production of phenolic off-flavours and over-attenuation leading to gushing [5,30]. This could explain the lower value of the apparent extract in the FM samples [30,31]. The most detected non-*saccharomyces* wild yeast was *Brettanomyces*. Its presence changes the organoleptic properties of a beer due to the production of secondary metabolites when performing alcoholic fermentation [32]. Undesirable flavours such as horse sweat, barnyard, medicinal or leathery could appear when beer is spoiled by this yeast species.

According to the literature [9,18,28], Gram-positive bacteria are the most threatening contaminants in the brewery due to their rapid growth rate and tolerance to high temperatures and low pH. The predominant Gram-positive catalase-negative bacteria that are beer spoilers are the lactic acid bacteria (LAB) *Lactobacillus* and *Pediococcus,* which are responsible for almost 70% of all spoilage incidents in brewing industries [9,18,33]. Based on microscopic analysis, the lactic acid bacteria that spoiled the samples could be *Lactobacillus.* These bacteria produce lactic acid that lowers the pH value of beer (often below 4.3), which could explain the lower pH value of the fresh microfiltered samples (around 4.0). *Lactobacillus* can also produce turbidity and off-flavours, making beer sour via lactic and acetic acid production, which explains why in the sensory descriptive test the panellists described turbidity, lactic acid and acidity/spicier in visual, and aroma/flavour and taste description, respectively, in the FM samples [5,33]. In Figure 5A,B we observed that, except for turbidity, which is more associated with the AM samples, all the other sensory descriptors mentioned above are positively associated with the FM samples. Aged and pasteurised samples did not have any bacterial or yeast spoilage. Since the membranes used in the microfiltration process are capable of retaining bacteria and yeasts, we can say that the source of contamination is located between the filter and the pasteurisation tunnel. According to Suiker [30], most cases of spoilage involving *S. diastaticus* are associated with contamination that occurs post-fermentation, most likely during filling, with biofilms being the main source of this contamination. Moreover, in a review written by Suzuki [5] on the emergence of new spoilage microorganisms in the brewing industry, the author reported *Lactobacillus linderi* as one of the lactic acid bacteria that represents a potential threat to unpasteurised beers, since it can more easily penetrate the sterile membrane filter due to its small size. 

The GC–MS results show that turbidity, mentioned as a sensory characteristic of the FM samples in sensory analysis, is positively correlated with the AM samples. According to Jaluska et al. [34], in their study on the influence of transport and storage conditions on beer quality and flavour, haze formation (turbidity) could be linked to an increase in temperature. Moreover, we observed that furfural (4) and 2,3-butanediol (6) were detected only in the AM samples. Furfural (4) presence in aged beer seems to be directly associated with sensory changes, namely flavour staling, and its increased levels seem to occur independently of oxygen concentration [26]. The chemometric analysis confirmed that both compounds are positively correlated with the AM samples. Furfural (4) is considered a heat indicator in beer and its presence almost invariably results from exposure of the beer to higher temperatures than the AM samples. According to Madigan et al. [35], the higher the temperature, the greater the formation of furfural, which explains the presence of this compound in the force-aged microfiltered samples. On the contrary, despite being subjected to high temperatures, furfural (4) was not detected in the matrix of the pasteurised samples, both FP and AP. The presence and absence of furfural (4) in the microfiltered and pasteurised samples can be explained by the difference in the way that temperature was applied to both samples. While in force-aged microfiltered samples (AM) the temperature increase occurred abruptly (from 10 °C to 55 °C in less than 1 h) and the samples were maintained at 55 °C for 6 days, in the pasteurised samples (FP and AP), the temperatures increased and decrease gradually in the pasteurised tunnel.

Regarding the presence of benzoic acid (12) and methyl benzoate (07) in all samples except AM, it was observed (in Figure 5A,B) that both compounds have a positive loading in LV2, being positively correlated with the FM, FP and AP samples. The correlation matrix in Figure S1 (Supplementary Materials) shows that these compounds are not associated with each other. 

## 5. Conclusions

In this paper, the influence of microfiltration and pasteurisation on the quality of beer during its shelf life was analysed using multivariate analysis.

Regarding the efficiency of the microfiltration and pasteurisation processes in inactivating spoilage contamination in beer, the microbiological analyses detected spoilage contamination from yeasts and bacteria in all FM samples. This can be concluded that in a non-sterile bottling process, applying only microfiltration as a preservation process may not be enough to guarantee the sterilisation of the product, since contamination may occur after this step due to the presence of contaminating microorganisms in the equipment or the air. To achieve colloidal, microbiological and flavour stability to guarantee beer quality during shelf life, the results of the analyses performed showed that applying only microfiltration as a conservation method in a non-sterile bottling process may not be enough to guarantee the sterilisation of the product and consequently its stability during shelf life. This is because contamination may happen after this step due to the presence of contaminating microorganisms in the equipment or the air. Since some microorganisms that spoil beer are resistant to the intrinsic hurdles in a non-sterile bottling process, applying pasteurisation as a preservation process is more efficient than just applying microfiltration. On the contrary, the samples that passed through the pasteurisation process (FP and AP) did not present any spoilage contaminants, which leads us to the conclusion that in a non-sterile bottling process, applying pasteurisation as a conservation process is more efficient than just applying microfiltration. 

It was observed that AM samples did not have any spoilage bacteria or yeast after the forced-ageing process. Based on this result, it can be said that the forced-age process used to accelerate the ageing of a beer is capable of inactivating some microorganisms that could influence the beer’s sensory quality profile; thus, we recommend further studies using natural ageing to evaluate the effects of spoilage contamination on the quality parameters of microfiltered samples during shelf life and compare them with pasteurised samples. 

In conclusion, despite some disadvantages presented above in the use of heat treatments to improve the stability of the product during its shelf life, pasteurisation is a conservation process recommended for its ability to inactivate the spoilage contaminants that are responsible of the rapid decline in beer quality during its shelf life. 

## Figures and Tables

**Table 1:** Treatments applied to the samples *.

Samples	Fresh	Forced Ageing
Microfiltered	34	34
Pasteurised	34	34
Total	68	68

* All beers were served at 10 °C during sensory analysis.

**Table 2:** Attributes evaluated in the sensory analysis.

Aspect Analysed	Descriptor
Visual	foam colour, beer colour, turbidity, foam size, Belgian lace and foam quality, scored from 1 to 10.
Aroma	alcoholic, sweetness, solvent, vegetables, species, nuts, toast, fruity, floral, biscuit, sulphurous, phenolic, aged, lactic acid, acetic acid, bread, butter. The panellists were asked to choose the aroma that best described the sample.
Flavour	alcoholic, sweetness, solvent, vegetables, species, nuts, toast, fruity, floral, biscuit, sulphurous, phenolic, aged, lactic acid, acetic acid, bread, butter. The panellists were asked to choose the flavour that best described the sample.
Taste	sweetness, bitterness, acidity, salty and umami, scored from 1 to 10.
Mouth sensation	body, carbonation, texture, alcohol, astringency, spicy, metallic, scored from 1 to 10.

**Table 3:** Results of the microbiological analyses. FM1, fresh microfiltered sample 1; AM, aged microfiltered sample; FP, fresh pasteurised sample; AP, aged pasteurised sample. * Spoilage was not detected in any sample.

Samples	Analysis	YM (*Saccharomyces* Yeast) CFU/10⁶ Brewing Yeast cells	LYSINE (non-*Saccharomyces* Yeast) CFU/10⁶ Brewing Yeast Cells	WL (Aerobic Incubation) cfu per 0.1 mL	WL (Anaerobic Incubation) CFU per 0.1 mL	Gram Staining Test	Catalase Test
FM1	1	1	5	>300/>300	>300/>300	+	-
	2	2	3	>300/>300	>300/>300	+	-
FM2	1	2	2	>300/>300	>300/>300	+	-
	2	1	2	>300/>300	>300/>300	+	-
FM3	1	1	1	>300/>300	>300/>300	+	-
	2	5	0/0	>300/>300	>300/>300	+	-
FM4	1	3	0/0	>300/>300	>300/>300	+	-
	2	5	4	>300/>300	>300/>300	+	-
FM5	1	2	2	>300/>300	>300/>300	+	-
	2	8	2	>300/>300	>300/>300	+	-
AM * FP * AP *	1	0/0	0/0	0/0	0/0	0/0	0/0
	2	0/0	0/0	0/0	0/0	0/0	0/0

**Table 4:** GC–MS compound identification in both microfiltered and pasteurised beer samples. FM, fresh microfiltered; AM, aged microfiltered; FP, fresh pasteurised; AP, aged pasteurised.

No.	Name	CAS	ODOR [17]	FM	AM	FP	AP
01	ethanol	64-17-5	sweet	X	X	X	X
02	2-methyl, 1-butanol	137-32-6	malt	X	X	X	X
03	3-methyl,1-butanol	123-51-3	whiskey, malt	X	X	X	X
04	furfural	98-01-1	bread, almond, sweet	-	X	-	-
05	acetic acid	64-19-7	sour	X	X	X	X
06	2,3-butanediol	513-85-9	creamy, buttery	-	X	-	-
07	methyl benzoate	93-58-3	prune, lettuce, herb, sweet	X	-	X	X
08	β-phenethyl acetate	103-45-7	rose, honey, tobacco	X	X	X	X
09	caproic acid	142-62-1	sweet	X	X	X	X
10	2-phenylethyl alcohol	60-12-8	honey, spice, rosa, lilac	X	X	X	X
11	octanoic acid	124-07-2	sweet, cheese, rancid	X	X	X	X
12	benzoic acid	65-85-0	urine	X	-	X	X

(X)—Presence of the compound in the samples; (-)—Absence of the compound in the sample.

**Table 5:** PLS2: Main variables responsible for determining the efficiency of the microfiltration and pasteurisation processes on the sensory quality.

GC–MS Analysis	Physicochemical Analysis	Sensory Analysis
2-methyl, 1-butanol (2)	IBU	Visual description
3-methyl,1-butanol (3)	VDK	Foam Quality
furfural (4)	OG	Foam Size
3-methyl,1-butanol (6)	Colour	Turbidity
Methyl benzoate (7)	pH	Aroma description
β-phenethyl acetate (8)		Lactic acid
2-phenylethyl alcohol (10)		flavour description
Octanoic acid (11)		Bread
Benzoic acid (12)		Biscuit
		taste description
		Acidity

## Data Availability

Data are contained within the article.

## Supplementary Materials

The following supporting information can be downloaded at: https://www.mdpi.com/article/10.3390/foods13010122/s1, Figure S1: Correlation matrix of the physicochemical parameters, GC-MS peaks and sensory attributes. Figure S2: Correlation loadings of the PLS2 model (X variables in blue and Y variables in red). Table S1: ANOVA results of the comparison of the physicochemical analysis for the different samples: FM—Fresh Microfiltered; AM—Aged Microfiltered; FP—Fresh Pasteurized; AP—Pasteurized. Table S2: ANOVA results of the comparison of GC-MS data for the different simples: FM—Fresh Microfiltered; AM—Aged Microfiltered; FP—Fresh Pasteurized; AP—Pasteurized.
