# Supplementary material for: Multivariate Analysis of the Influence of Microfiltration and Pasteurisation on the Quality of Beer during Its Shelf Life
Source: Foods. 2023 Dec 29;13(1):122. doi: 10.3390/foods13010122 (PMC10778496; doi:10.3390/foods13010122)
Supplement: Supplementary file 1 [file foods-13-00122-s001.zip › foods-2727245-supplementary.pdf]

Supplementary Material

Table S1. ANOVA results of the comparison of the physicochemical analysis for the different samples: FM – Fresh Microfiltered: AM – Aged Microfiltered; FP – Fresh Pasteurized; AP -Pasteurized.

**Samples**	Repetitions	Apparent Extract		Bi eness - IBU		Colour	
		Mean*	standard deviation	Mean*	standard deviation	Mean*	standard deviation
**AM**	5	2,0^{a}	0,07	13,49^{b}	0,32	9,40^{b}	0,89
**FM**	5	1,62^{b}	0,081	13,55^{b}	0,13	13,40^{a}	0,55
**AP**	5	1,92^{a}	0,04	15,33^{a}	0,37	7,95^{c}	0,08
**FP**	5	1,94^{a}	0,11	15,24^{a}	0,90	8,00^{c}	0

**Samples**	Repetitions	pH		VDK	
		Mean*	standard deviation	Mean*	standard deviation
**AM**	5	4,30^{b}	0	0,64^{b}	0,11
**FM**	5	3,90^{c}	0	0,28^{c}	0,04
**AP**	5	4,56^{ab}	0,05	1,40^{a}	0,07
**FP**	5	4,58^{a}	0,08	1,30^{a}	0,16

*By column, means with same letter are not signifficantly different according to Fisher test ($p < 0,05$).

Table S2. ANOVA results of the comparison of GC-MS data for the different simples: FM – Fresh Microfiltered: AM – Aged Microfiltered; FP – Fresh Pasteurized; AP -Pasteurized.

**Samples**	Repetitions	2-methyl, 1-butanol		3-methyl, 1-butanol		acetic acid	
		Mean*	standard deviation	Mean*	standard deviation	Mean*	standard deviation
**AM**	5	10645723[a]	425690	69872988 [a]	6845601	4361536 [a]	3040205
**FM**	5	8378586[ab]	951401	57915673 [ab]	8620330	4017503 [a]	991809
**AP**	5	6753296[b]	3137184	53411466 [b]	10064468	3431667 [a]	1633763
**FP**	4	7931926[ab]	2759565	59762112 [ab]	11268427	1826468 [a]	128794
**Samples**	Repetitio	methyl benzoate		β-phenethyl acetate		caproic acid	
		Mean*	standard deviation	Mean*	standard deviation	Mean*	standard deviation
**AM**	5	n.d.	-	363159 [b]	117157	304043[c]	42330
**FM**	5	15338787 [b]	7288922	322563 [b]	52044	747808 [a]	77081
**AP**	5	27232921 [a]	4267484	631270 [a]	101789	585589 [b]	64394
**FP**	4	21387463 [ab]	2492559	509323 [a]	66059	696037 [a]	53493
**Samples**	Repetitions	2-phenylethyl alcohol		Octanoic acid		Benzoic acid	
		Mean*	standard deviation	Mean*	standard deviation	Mean*	standard deviation
**AM**	5	4729680[b]	1217907	431522 [c]	93969	n.d.	-
**FM**	5	5684452 [ab]	1006138	2060345 [a]	698630	3274700 [a]	1837820
**AP**	5	6348493 [a]	485698	1057990 [b]	109607	6246513 [a]	3643163
**FP**	4	6083649 [a]	729478	509323 [b]	108382	3008385 [a]	287580

*By column, means with same letter are not signifficantly different according to Fisher test (p < 0,05).

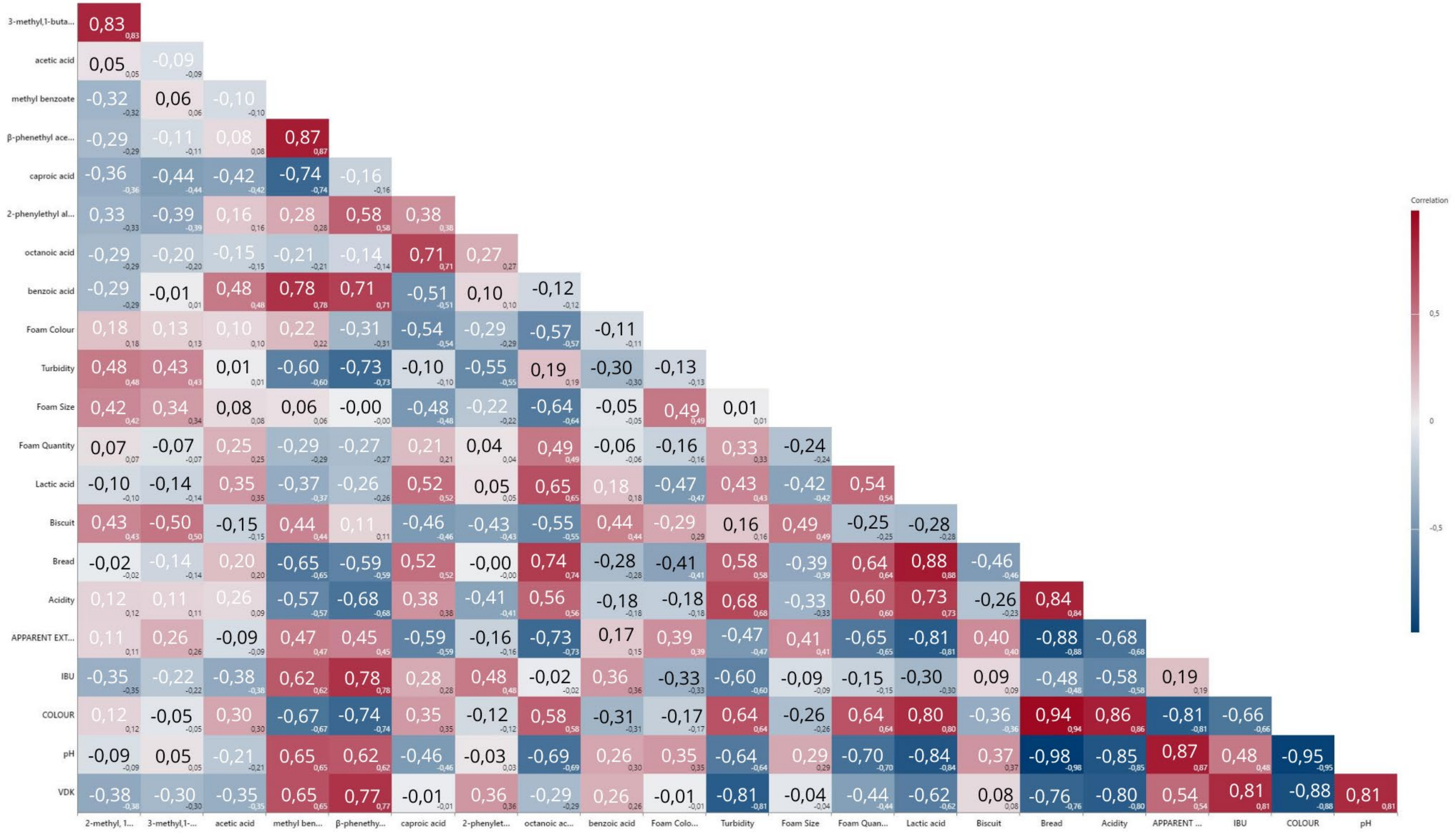

Figure S1. Correlation matrix of the physicochemical parameters, GC-MS peaks and sensory attributes.

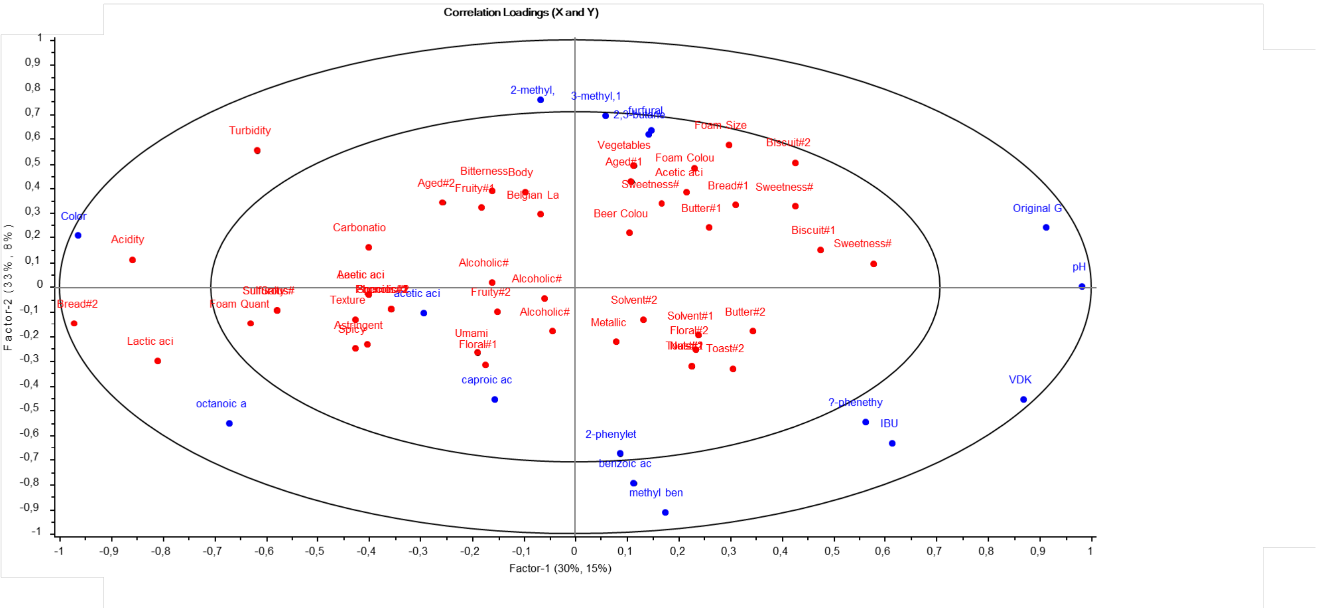

Figure S2. Correlation loadings of the PLS2 model (X variables in blue and Y variables in red).
